# Measuring mentalizing: A comparison of scoring methods for the hinting task

**DOI:** 10.1002/mpr.1827

**Published:** 2020-05-09

**Authors:** Hans S. Klein, Cassi R. Springfield, Emily Bass, Kelsey Ludwig, David L. Penn, Philip D. Harvey, Amy E. Pinkham

**Affiliations:** ^1^ School of Behavioral and Brain Sciences The University of Texas at Dallas Richardson Texas USA; ^2^ Department of Psychology Indiana University–Purdue University Indianapolis Indianapolis Indiana USA; ^3^ Department of Psychology and Neuroscience University of North Carolina‐Chapel Hill Chapel Hill North Carolina USA; ^4^ School of Behavioural and Health Sciences Australian Catholic University Melbourne Victoria Australia; ^5^ Department of Psychiatry and Behavioral Sciences University of Miami Miller School of Medicine Miami Florida USA; ^6^ Research Service Miami VA Healthcare System Miami Florida USA; ^7^ Department of Psychiatry University of Texas Southwestern Medical School Dallas Texas USA

**Keywords:** methodology, scale validation, schizophrenia, SCOPE scoring, theory of mind

## Abstract

**Objective:**

The Social Cognition Psychometric Evaluation (SCOPE) study supported the utility and practicality of the Hinting task as a measure of social cognition/mentalizing in clinical trials, specifically with the SCOPE authors' stringent scoring system. However, it remains unclear whether the SCOPE scoring system is necessary for the task to be judged as psychometrically sound.

**Method:**

Independent raters rescored data from the three phases of SCOPE using the Hinting task's original scoring criteria. Psychometric properties of the task when scored with the original criteria versus more stringent SCOPE criteria were compared in a large sample of individuals with chronic schizophrenia (*n* = 397) and matched controls (*n* = 300) as well as a smaller sample of individuals with early psychosis (*n* = 38) and controls (*n* = 39).

**Results:**

In both samples, SCOPE criteria resulted in lowered average scores and reduced ceiling effects. Further, revised scoring resulted in strengthened relationships between the hinting task and outcome measures in the chronic sample, and better differentiated early psychosis patients from controls. Conversely, test‐retest reliability and internal consistency estimates were not improved using revised scoring and remained suboptimal, particularly for healthy controls.

**Conclusion:**

Overall, SCOPE scoring criteria improved some psychometric properties and clinical utility, suggesting that these criteria should be considered for implementation.

## 1 INTRODUCTION

Mentalizing/Theory of Mind (ToM), or the ability to interpret and infer another's thoughts or intentions, is a necessary skill when interacting with others in a social world. Impairments in ToM may impede successful navigation of social situations and is related to failures in adaptive social functioning (Fett et al., 2011; Halverson et al., 2019). Prior research suggests ToM is significantly impacted in a number of psychiatric disorders, including autism spectrum disorders (1Morrison et al., 2019), depression (Bora & Berk, 2016; Cusi, Nazarov, MacQueen, & McKinnon, 2013), bipolar disorder (Bora, Yucel, & Pantelis, 2009a; McKinnon, Cusi, & MacQueen, 2010), social anxiety disorder (Hezel & McNally, 2014; Washburn, Wilson, Roes, Rnic, & Harkness, 2016), Parkinson's Disease (Kosutzka et al., 2019), and moderate‐to‐severe traumatic brain injury (Tousignant et al., 2018). Additionally, ToM deficits have been identified as a trait marker for liability to psychosis, presenting early in psychotic disorders (Bertrand, Sutton, Achim, Malla, & Lepage, 2007; Ludwig, Pinkham, Harvey, Kelsven, & Penn, 2017), in comorbid diagnoses (Wang, Wang, Chen, Zhu, & Wang, 2008), and in healthy first‐degree relatives (Versimissen et al., 2008). For individuals with schizophrenia, ToM is largely impaired when compared to healthy populations (Bora, Yucel, & Pantelis, 2009b, *d* = .08 for remitted patients and *d* = 1.21 for nonremitted patients; Sprong, Schothorst, Vos, Hox, & Engeland, 2007, *d* = 1.26). Further, mentalizing ability in schizophrenia predicts social skills and behavior in this population (Halverson et al., 2019).

The Hinting Task (Corcoran, Mercer, & Frith, 1995) is one of the most widely used assessments for measuring mentalizing abilities in patients with schizophrenia, and has been administered to individuals diagnosed with autism spectrum disorders (Morrison et al., 2019), Parkinson's Disease (Kosutzka et al., 2019), and moderate‐to‐severe traumatic brain injury (Tousignant et al., 2018). Although this task was designed to measure deficits in clinical populations via 10 vignettes assessing an individual's ability to infer intent from indirect speech, it has been criticized for its poor psychometric properties (Davidson, Lesser, Parente, & Fiszdon, 2018; Mallawaarachchi, Cotton, Anderson, Killackey, & Allott, 2019). Specifically, this task has demonstrated ceiling effects in both patients with schizophrenia spectrum disorders (Lindgren et al., 2018; Marjoram et al., 2006; Roberts & Penn, 2009; Versimissen et al., 2008) and healthy controls (Corcoran & Frith, 2003; Corcoran & Frith, 2005), indicating that this measure may underestimate or inaccurately reflect true mentalizing abilities in clinical and nonclinical samples. In one psychosocial treatment study, Roberts and Penn (2009) found that over half of participants (57%) scored at normative levels on the task at baseline (a score of 17 or above out of 20), potentially limiting the ability to observe improvement in subsequent measurements.

Despite these limitations, the Hinting Task was selected for consideration in the Social Cognition Psychometric Evaluation (SCOPE) study, which sought to identify the best available measures of social cognition for use in clinical trials of schizophrenia spectrum illnesses (Pinkham et al., 2014; Pinkham, Penn, Green, & Harvey, 2016). Results from the final validation phase of SCOPE supported the utility and practicality of the Hinting task in clinical research (Pinkham, Harvey, & Penn, 2018) stating that, in contrast to the aforementioned criticisms of the task, this measure demonstrated limited floor and ceiling effects in patients and healthy controls (less than 7% of the total sample). Notably, the authors of SCOPE developed and utilized a novel, more stringent scoring system for the Hinting task throughout SCOPE, which may have resulted in improved psychometric properties over the original scoring method (Pinkham et al., 2016). Findings from SCOPE suggest that the Hinting task demonstrated small practice effects (patients: initial phase *d*_*z*_ = .19; final validation study *d*_*z*_ .15; healthy controls: initial phase *d*_*z*_ = .31; final validation study *d*_*z*_ .18) and adequate test‐retest reliability in patients (initial phase r = .639; final validation study *r* = .695), with slightly lower test‐retest reliability in healthy controls (initial phase r = 424; final validation study *r* = .509) (Pinkham et al., 2016; Pinkham et al., 2018). Further, the Hinting task was identified as a significant predictor of real‐world outcomes, including functional capacity, social competence, social functioning, and community‐living skills (Pinkham et al., 2016; Pinkham et al., 2018).

Building upon literature that suggests that length of illness may impact social cognitive ability, Ludwig et al. (2017) investigated whether the utility and practicality of social cognitive tasks utilized in the primary SCOPE study extended to younger individuals with first episode psychosis (FEP). Within their sample of individuals with FEP (*M*_age_ = 23.45), the authors noted that the Hinting task and revised scoring system showed good test‐retest reliability (*r* = .74) and limited practice effects (*d*_*z*_ = .41–.64) in FEP (Ludwig et al., 2017). Further, only two patients (<6% of the sample) showed floor/ceiling effects. Consistent with the results of SCOPE, the Hinting task demonstrated sound psychometric properties and was shown to be a significant predictor of real‐world outcomes for individuals early in the course of illness when the more stringent scoring method developed by the SCOPE authors was utilized.

The reported results from the SCOPE study highlight that the Hinting task is appropriate for use in patients with psychosis, regardless of stage of illness when utilizing a more stringent scoring system. However, it is unclear how these psychometric properties compare to the original scoring criteria and whether the more stringent criteria are necessary and warrant widespread adoption. The current study therefore used all available SCOPE data to compare the psychometric properties of the Hinting task when scored with the SCOPE system to those obtained with the original scoring criteria in both chronic and FEP. We hypothesized that the revised SCOPE scoring criteria would result in overall improved psychometric properties of the Hinting Task, specifically higher estimates of test‐retest reliability and internal consistency. By reducing the overall number of participants scoring at ceiling, we also anticipated that the SCOPE scoring system would introduce more variability within the sample, and result in stronger associations with functional outcome measures.

## 2 METHODS

### 2.1 Participants

Collapsing across the three SCOPE phases resulted in a sample of 790 unique participants. The sample was then divided into either patients with chronic schizophrenia spectrum diagnoses and matched healthy controls, or early psychosis patients and their matched healthy controls. Sixteen participants, 12 patients and four healthy controls, were omitted from the chronic analyses due to being extreme outliners (−3 SD) with either the original or SCOPE scoring systems. This resulted in final sample sizes of 697 participants in the “chronic” analyses (397 patients with schizophrenia spectrum diagnoses and 300 healthy controls) and 77 participants in the “early psychosis” analyses (38 patients with schizophrenia spectrum diagnoses and 39 healthy controls). Demographic information for both samples is provided in Table 1, and results will be discussed for each of these groups individually below.

**TABLE 1:** Participant demographic and clinical characteristics

	Chronic sample		Early psychosis sample	
	Patients (*n* = 397)	Controls (*n* = 300)	Patients (*n* = 38)	Controls (*n* = 39)
	*n* (%)	*n* (%)	*n* (%)	*n* (%)
Male*	261 (65.7)	170 (56.7)	33 (86.8)	32 (82.1)
Race				
Caucasian	185 (46.6)	137(45.7)	28 (73.7)	26 (66.7)
African American	186 (46.9)	144 (48.0)	4 (10.5)	5 (12.8)
Asian	8 (2.0)	10 (3.3)	2 (5.3)	2 (5.1)
Other	18 (4.5)	9 (3.0)	4 (10.5)	6 (15.4)
Ethnicity				
Hispanic	72 (18.1)	56 (18.7)	2 (5.3)	6 (15.4)
Non‐Hispanic	325 (81.9)	244 (81.3)	36 (94.7)	33 (84.6)

	M (*SD*)	M (*SD*)	M (*SD*)	M (*SD*)
Age	41.90 (11.81)	41.15 (12.53)	23.45 (3.01)	23.77 (3.39)
Education (years)*	12.92 (2.36)	13.94 (1.86)	14.03 (1.52)	15.44 (1.80)
WRAT‐3*	94.61 (14.96)	98.85 (12.36)	105.87 (9.35)	107.82 (8.91)
UPSA‐B	70.40 (14.10)	—	70.55 (11.63)	—
SSPA‐Avg	4.16 (0.50)	—	4.15 (0.40)	—
SLOFinf‐Avg	4.05 (0.59)	—	4.08 (0.63)	—
SLOFsr‐Avg	4.16 (0.55)	—	4.25 (0.46)	—
PANSS (initial visit)				
Positive total	16.12 (5.40)	—	17.53 (4.91)	—
Negative total	13.81 (5.20)	—	16.58 (3.96)	—
General total	31.38 (8.01)	—	36.00 (5.95)	—
PANSS (follow‐up)				
Positive total	15.55 (5.03)	—	15.44 (4.07)	—
Negative total	13.90 (5.21)	—	16.06 (4.35)	—
General total	30.34 (7.54)	—	34.75 (6.69)	—

* Chronic sample patient and controls differed on gender, *X* ^*2*^(1) = 5.965, *p* = .018, years of education, *t*(694.01) = 6.418, *p* < .001, *d* = 0.472, and WRAT‐3, *t*(689.50) = 4.093, *p* < .001, *d* = 0.305. Early psychosis sample patient and controls differed on years of education only, *t*(75) = 3.717, *p* < .001, *d* = 0.845.

Abbreviations: PANSS, Positive and Negative Syndrome Scale; SCOPE, Social Cognition Psychometric Evaluation; SLOFinf, Specific Level of Functioning Scale–informant report; SLOFsr, Specific Level of Functioning Scale–self report; SSPA, Social Skills Performance Assessment; UPSA, UCSD Performance‐Based Skills Assessment; WRAT‐3, Wide Range Achievement Test.

### 2.2 Measures

#### 2.2.1 Hinting task and scoring criteria

During administration of the Hinting task (Corcoran et al., 1995), the rater reads aloud a short vignette describing an interaction between two characters. Each of the 10 passages end with one character dropping a hint, and participants are asked to indicate what the character truly meant. If the participant is inaccurate in their assessment of the character's intent, the rater provides a second hint, allowing the participant to receive partial credit. In the SCOPE administration of the task, participants could ask for the vignette or additional hints to be read again as needed; however, no additional queries were administered to elicit more detailed responses. Individual items are scored from 0 to 2, with a “2” indicating perfect understanding of the intention of the character in the scene, a “1” indicating partial credit, and a “0” indicating failure to infer intention. Performance is indexed as a total score that can range from 0 to 20.

The original scoring criteria are broad and allow a wide range of responses to earn full credit. The stringent scoring criteria developed by SCOPE authors and implemented throughout the phases of SCOPE attempts to reduce ambiguity when assessing the completeness of a participant's response in real time. For example, in the vignette, “Patsy is getting off the train with three heavy suitcases. John is standing behind her. Patsy says to John, ‘Gosh! These suitcases are a nuisance,’” the participant is asked to infer the intent of Patsy's statement. In the original scoring method, any response indicating that Patsy would like help with her suitcases earns full credit. The SCOPE criteria, however, emphasize that in each of the vignettes, a direct request is being made of the second character in the scenario. In the example above, a correct answer must identify not only that Patsy needs help with her suitcases but also that she wants John, specifically, to help carry them. Correct answers are required to include the intention of the person as well as a request of the other person in the scenario. Furthermore, the SCOPE authors identified key phrases or words that assist in reducing variability in rater scoring. These revised scoring criteria are provided in Appendix S1***.

#### 2.2.2 Functional outcome measures

Data for functional outcomes from the SCOPE study were utilized to assess the relationship between Hinting task scoring methods and both informant report and performance‐based functioning. Social competence was assessed with the Social Skills Performance Assessment (SSPA; Patterson, Moscona, McKibbin, Davidson, & Jeste, 2001), and functional capacity was assessed using the UCSD Performance‐Based Skills Assessment, Brief (UPSA‐B; Mausbach, Harvey, Goldman, Jeste, & Patterson, 2007). Both an informant‐rated version and self‐reported version of the Specific Level of Functioning Scale (SLOF, Schneider & Struening, 1983) were used to gauge real‐world functioning. Informants were identified by participants and were either high contact clinicians, family members, or close friends with knowledge of the participant's daily functioning. The relationship between hinting scoring methods and functional outcomes was only assessed in the patient samples.

#### 2.2.3 Neurocognition and symptom assessment

As part of the SCOPE protocol, premorbid IQ was estimated at the participant's initial visit using The Wide Range Achievement Test (WRAT‐3) reading subscale (Wilkinson, 1993). Current neurocognitive abilities were assessed with an abbreviated version of the MATRICS consensus cognitive battery (MCCB; Nuechterlein et al., 2008). Additionally, symptom severity was measured in patients at each visit via the Positive and Negative Syndrome Scale (PANSS; Kay, Fiszbein, & Opler, 1987).

### 2.3 Procedures

This study utilized data from the three phases of the SCOPE study, in which participants completed a comprehensive social cognitive battery at two time points, approximately 2–4 weeks apart. Information regarding participant recruitment and administration procedures for the SCOPE study have previously been reported (Pinkham et al., 2016; Pinkham et al., 2018). Hinting task responses were recorded verbatim and scored in real time using the stricter, revised SCOPE criteria. Given the availability of these verbatim responses, independent raters that were not previously trained on the SCOPE scoring criteria were able to review all participant responses and rescore them, assessing whether provided responses were sufficient for full or partial credit based upon the original criteria. Three independent raters were trained to good reliability, *ICC* (1,3) = 0.840, based upon the original scoring guidelines provided by Corcoran et al. (1995).

### 2.4 Statistical analyses

Analyses followed the statistical plan used in the original SCOPE study, and psychometric properties for the chronic and early psychosis groups were analyzed separately. For the chronic sample, distributions of the hinting task scores were first assessed for both the original and SCOPE scoring criteria. Outliers, defined as −3 *SD* from the mean for either scoring system, were excluded from analyses (n = 12 patients, four controls). Test‐retest reliability was computed using Pearson's *r* correlation coefficients whereas internal consistency was evaluated via Cronbach's alpha. Practice effects (paired‐samples *t*‐tests with Cohen's *d*_*z*_) and floor/ceiling effects (number of participants scoring at 0 or scoring 100%) were assessed to determine utility as a repeated measure. Additionally, although we define ceiling effects as perfect performance (scoring 20 out of 20), it is also important to consider whether the task allows room for improvement in clinical trials (Murthy, Xu, Zhong, & Harvey, 2019). We therefore also report the number of participants achieving near‐perfect scores at initial testing (≥ 17 out of 20) using each scoring method. Finally, to examine relationship to functional outcomes, Pearson's *r* correlations were calculated with the Hinting task score from the participant's initial visit. Partial correlations between Hinting scores and outcomes while controlling for MCCB neurocognitive performance were also calculated.

Independent sample *t*‐tests with Cohen's *d* examined group differences between patients and controls, and paired *t* test were used to examine mean differences between the original scoring criteria and SCOPE scoring. Fischer's *z* was used to compare test‐retest reliability indices between scoring criteria, as well as the relationships between scoring criteria and functional outcomes. Feldt tests (Feldt, Woodruff, & Salih, 1987) were performed to compare estimates of internal consistency between scoring criteria. As the current study collapses samples across the three phases of the SCOPE study to form the chronic sample, current results differ slightly from previously published reports. Appendix S1 provides more direct comparisons of individual samples from each of the previously published SCOPE psychometric papers with rescored results.

For the early psychosis sample, analyses were similar; however, as the early psychosis sample was considerably smaller, analyses were run both with and without outliers. Two participants were identified as outliers (one patient and one healthy control); however, their removal from the data did not significantly impact the pattern of results. Therefore, results below are reported with outliers included, although analyses excluding these three participants can be found in the Appendix S1.

## 3 RESULTS

### 3.1 Test‐retest reliability

#### 3.1.1 Chronic sample

Although test‐retest reliability estimates as assessed by both the original and SCOPE scoring criteria were within the benchmark standards for acceptability (*r* ≥ .6; Kraemer, Kupfer, Clarke, Narrow, & Regier, 2012) for the patient sample, we observed a significant decrease in test‐retest reliability when using the SCOPE scoring system compared to the original criteria. Scoring system did not significantly impact the test‐retest reliability for healthy controls; however, both scoring systems resulted in test‐retest reliability estimates below the benchmark standards for healthy controls (Table 2).

**TABLE 2:** Test‐retest reliability and internal consistency

	Chronic subset					
	Test‐retest reliability (Pearson *r*)		Internal consistency (Cronbach's alpha)			
Task	Patients (*n* = 375)	Controls (*n* = 286)	Patients T1 (*n* = 395)	Patients T2 (*n* = 377)	Controls T1 (*n* = 299)	Controls T2 (*n* = 287)
Hinting (*SCOPE scoring*)	.604	.549	.629	.679	.553	.569
Hinting (*original scoring*)	.712	.584	.686	.692	.526	.520
Significance test^a^	3.340***	0.744	3.197	0.190	0.285	0.938

	Early psychosis subset					
Task	Patients (*n* = 36)	Controls (*n* = 36)	Patients T1 (*n* = 38)	Patients T2 (*n* = 36)	Controls T1 (*n* = 39)	Controls T2 (*n* = 36)
Hinting (*SCOPE scoring*)	.735	.204	.685	.513	.493	.528
Hinting (o*riginal scoring*)	.613	.360	.672	.561	.093	.653
Significance test^a^	1.937	−1.292	0.046	0.189	4.581*	2.425

*Note:* *p < .05, ***p < .001.

a Fisher's z was calculated to compare test‐retest reliability estimates. Feldt tests were performed to compare estimates of internal consistency.

Abbreviation: SCOPE, Social Cognition Psychometric Evaluation.

#### 3.1.2 Early psychosis sample

Similar to the chronic sample, test‐retest reliability as assessed by both the original and SCOPE scoring criteria was within benchmark standards for the patient sample, but below standards for healthy controls. Scoring criteria did not significantly impact estimates of test‐retest reliability for either group within this sample.

### 3.2 Internal consistency

#### 3.2.1 Chronic sample

Internal consistency was calculated for both initial and follow‐up task administrations for both groups (Table 2). Across the sample, these scores did not reach recommended levels for internal consistency (*α* = .80; Nunally, 1967), and scoring criteria did not significantly impact estimates of internal consistency for either group at any time point.

#### 3.2.2 Early psychosis sample

Similar to the chronic sample, internal consistency for each time point was below acceptable levels for both groups across all time points. We observed that the internal consistency estimate for healthy controls at time 1 is extremely low when using the original scoring criteria, which could be a result of limited variability within the item scores for this group. SCOPE scoring significantly improved this estimate, though it still does not meet the acceptable thresholds for estimates of internal consistency.

### 3.3 Utility as a repeated measure and direct comparisons of scoring criteria

#### 3.3.1 Chronic sample

Across groups, participants significantly improved their performance from initial visit to follow‐up when using either the original or SCOPE scoring criteria (Table 3), but these practice effects were small when using either scoring criteria (original *d* = .181 and revised *d* = .224). When directly comparing scoring methods, mean scores at each time point were significantly reduced for both patients and healthy controls when utilizing the SCOPE scoring criteria (descriptive statistics have been provided in Table 3, statistical comparisons can be found in Table 4). The total number of participants scoring at ceiling were greatly reduced when using the SCOPE scoring criteria, reducing the total number from 58 participants (20% of sample) to 15 (5%) in the control group at the initial visit and from 80 (28%) to 22 (8%) at retest. For the patient sample, the overall number of participants receiving a perfect score was reduced from 30 participants (8% of sample) to 3 (0.8%) at the initial visit and from 55 (15%) to 6 (1.6%) at follow‐up. In considering near‐perfect performance and the benefits of capturing potential improvements resulting from treatment, SCOPE scoring reduced the total number of those with near‐perfect scores (≥ 17 out of 20) at the initial visit from 189 (original scoring) to 90 chronic patients and from 235 to 135 healthy controls.

**TABLE 3:** Utility as a repeated measure

	Chronic												
	T_1_			T_2_			T_2_ − T_1_ difference		Number at floor/ceiling				
Task	Mean	*SD*	Skew/kurtosis	Mean	*SD*	Skew/kurtosis	Mean	*SD*	T_1_	T_2_	*t*	*p*	Cohen's *d*_z_
Patients													
Hinting (*n* = 375) (*SCOPE scoring*)	13.68	3.43	−.538/−.399	14.34	3.48	−.693/−.135	0.66	3.08	0/3	0/6	4.146	<.001	0.214
Hinting (*n* = 375) (*original scoring*)	15.66	3.40	−.863/−.061	16.13	3.37	−.876/−.025	0.46	2.56	0/30	0/55	3.504	.001	0.181
Controls													
Hinting (*n* = 286) (*SCOPE scoring*)	16.02	2.51	−.936/1.481	16.56	2.48	−1.190/1.956	0.53	2.37	0/15	0/22	3.792	<.001	0.224
Hinting (*n* = 286) (*original scoring*)	17.91	2.04	−1.730/3.837	18.30	1.88	−1.789/3.656	0.39	1.79	0/58	0/80	3.698	<.001	0.219

Early psychosis													
Task	Mean	*SD*	Skew/kurtosis	Mean	*SD*	Skew/kurtosis	Mean	*SD*	T_1_	T_2_	*t*	*p*	Cohen's *d*_*z*_
Patients													
Hinting (*n* = 36) (*SCOPE scoring*)	15.83	2.87	−1.172/1.633	17.08	2.13	−1.299/1.972	1.25	1.95	0/1	0/2	3.851	<.001	0.642
Hinting (*n* = 36) (*original scoring*)	17.69	2.42	−2.106/5.957	18.44	1.75	−1.755/4.087	0.75	1.93	0/5	0/11	2.328	.026	0.388
Controls													
Hinting (*n* = 36) (*SCOPE scoring*)	17.92	1.54	−1.051/1.612	18.00	1.64	−2.639/9.581	0.08	2.01	0/4	0/3	0.249	.805	0.042
Hinting (*n* = 36) (*original scoring*)	18.72	1.09	−.825/.489	18.83	1.56	−3.831/18.626	0.11	1.55	0/9	0/10	0.431	.669	0.072

Abbreviation: SCOPE, Social Cognition Psychometric Evaluation.

**TABLE 4:** Paired samples statistical test comparing Hinting task scoring methods

	Chronic sample				
Task	Original *M* (*SD*)	SCOPE *M* (*SD*)	*t* (*paired samples*)	*p*	Cohen's *d*_*z*_
Patients					
Hinting T1 (*n* = 395)	15.63 (3.41)	13.64 (3.44)	12.350	<.001	0.621
Hinting T2 (*n* = 377)	16.10 (3.39)	14.31 (3.50)	11.259	<.001	0.580
Controls					
Hinting T1 (*n* = 299)	17.92 (2.02)	16.02 (2.51)	14.063	<.001	0.813
Hinting T2 (*n* = 287)	18.30 (1.88)	16.56 (2.48)	13.233	<.001	0.781

	Early psychosis sample				
Task	Original *M*(*SD*)	SCOPE *M*(*SD*)	*t* (*paired samples*)	*p*	Cohen's *d*_*z*_
Patients					
Hinting T1 (*n* = 38)	17.61 (2.40)	15.82 (2.82)	7.487	<.001	1.215
Hinting T2 (*n* = 36)	18.44 (1.75)	17.08 (2.13)	5.846	<.001	0.974
Controls					
Hinting T1 (*n* = 39)	18.69 (1.06)	17.72 (1.78)	4.583	<.001	0.734
Hinting T2 (*n* = 36)	18.83 (1.56)	18.00 (1.64)	5.493	<.001	0.916

Abbreviation: SCOPE, Social Cognition Psychometric Evaluation.

#### 3.3.2 Early psychosis sample

For FEP patients, performance on the task significantly improved with repeated testing, noting small practice effects with the original criteria and moderate effects with the SCOPE scoring criteria. Healthy controls, however, did not demonstrate any significant practice effect for either the original or SCOPE scoring criteria. When directly comparing scoring methods for the early psychosis sample, mean scores at each time point were significantly reduced for both patients and healthy controls when utilizing the SCOPE scoring criteria (Table 4). Similar to the chronic sample, SCOPE scoring criteria greatly reduced the number of participants scoring at ceiling, reducing the total number for healthy controls at the initial visit from 9 (25% of the sample) to 4(11%), and from 10 (29%) to 3 (8%) at follow‐up. SCOPE criteria reduced the total number of patients scoring at ceiling from 5 (16% of sample) to 1 (3%) at the initial visit, and from 11 (31%) to 2 (6%) at follow‐up. The number of individuals scoring near‐perfect also decreased with SCOPE scoring, from 29 to 19 for patients and from 34 to 30 for healthy controls.

### 3.4 Relationship to functional outcomes

#### 3.4.1 Chronic sample

Correlations between measures of functioning and both original and SCOPE scoring of the hinting task can be found in Table 5. For the chronic patient population, small but significant correlations were observed between performance on the Hinting task and performance measures of functional capacity (UPSA), social competence (SSPA), and the informant rated measure of daily functioning (SLOF_Informant_). SCOPE scoring significantly increased the relationship between hinting and functional capacity.

**TABLE 5:** Correlations between initial visit Hinting scores and functional outcome measures in patients

	UPSA Total	SSPA average	SLOF informant	SLOF self‐report
Chronic subset	*n* = 384	*n* = 387	*n* = 320	*n* = 147
Hinting (*SCOPE scoring*)	.380*** (.336***)	.303*** (.124)	.160** (.206^a^)	.035 (.011)
Hinting (*original scoring*)	.276*** (.288**)	.261*** (.100)	.134* (−.020)	−.044 (−.006)
Fisher's z	2.346* (1.069)	.927 (.507)	.502 (4.461***)	1.018 (.218)
Neurocognitive				
Trails A	−.263***	−.122*	−.154**	−.123
Symbol coding	.352***	.283***	.206***	.041
HVLT‐R	.403***	.285***	.197***	.090
Letter‐number span	.499***	.285***	.215***	.124
Animal naming	.185***	.138**	.088	.001

Early psychosis subset	*n* = 38	*n* = 38	*n* = 30	*n* = 38
Hinting (*SCOPE scoring*)	.372* (.300)	.452** (.387)	−.234 (−.188)	.189 (.370)
Hinting (*original scoring*)	.404* (.311)	.451** (.339)	−.251 (−.219)	.090 (.325)
Fisher's z	−.381 (−.126)	.012 (.566)	.168 (.303)	1.107 (.527)
Neurocognitive				
Trails A	.266	−.251	−.086	−.130
Symbol coding	.265	.206	.157	.313
HVLT‐R	.311	−.009	.115	−.093
Letter‐number span	.559***	.179	−.099	.173
Animal naming	.227	.274	−.233	.288

*Note:* Correlations listed in parentheses are partial correlations between initial visit Hinting scores and functional outcome measures in patients after controlling for neurocognitive ability as measured by MATRICS consensus cognitive battery (MCCB) subscales. Fisher's z calculated to compare the effect of scoring criterion on correlations with functional outcome measures.

*Note:* *p ≤ .05; ** *p* ≤ .01; *** *p* ≤ .001.

Abbreviation: SCOPE, Social Cognition Psychometric Evaluation; SLOF, Specific Level of Functioning Scale; UPSA, UCSD Performance‐Based Skills Assessment.

We also observed significant correlations between neurocognitive ability and functional outcomes. Thus, in order to assess the unique relationship between social cognitive ability and functional outcome measures, we calculated partial correlations between hinting scoring and functional outcome measures, including all neurocognitive subscales as covariates. The small but significant relationship between hinting performance and functional capacity was retained after introducing covariates, though scoring system no longer significantly impacted this relationship. The relationship between hinting performance and social competence was no longer significant after controlling for neurocognitive ability. We observed a significant impact of the scoring system on the small, but significant, relationship between hinting performance and informant ratings of daily functioning after controlling for neurocognitive ability with the SCOPE criteria revealing a stronger relationship. No significant correlations were found between the Hinting task scores or neurocognitive subscales and the self‐reported measure of daily functioning (SLOF_Self‐report_) in the chronic sample. Correlations between neurocognitive subscales and Hinting scores are presented in the Appendix S1.

#### 3.4.2 Early psychosis sample

Performance on the Hinting task was significantly related to functional capacity (UPSA) and social competence (SSPA) when using both the SCOPE and original scoring criteria, though these were small effects. Unlike the chronic sample, Hinting task scores were not significantly related to informant rated daily functioning (SLOF_Informant_). Scoring criteria did not have any significant impact on these analyses, suggesting comparable relationships to functioning across scoring methods for this sample. We did not observe significant relationships between functional outcome measures and neurocognitive ability, with one exception of letter–number span (a measure of working memory) on functional capacity. Nevertheless, when adding neurocognitive ability as a covariate in correlational analyses, the partial correlations between hinting performance and functional outcome were no longer statistically significant for this small sample.

### 3.5 Group differences

#### 3.5.1 Chronic sample

Direct comparisons between patients and healthy controls are presented in Table 6. The groups significantly differed on task performance using the original scoring criteria at visits 1 and 2, with patients scoring lower than healthy controls at both time points. The updated SCOPE scoring retained these group differences with very comparable effect sizes.

**TABLE 6:** Group differences on Hinting task

Task	Patients		Controls				
Chronic sample	*n*	*M* (*SD*)	*n*	*M* (*SD*)	*t*	*p*	Cohen's *d*
SCOPE scoring							
Hinting T1	395	13.64 (3.44)	299	16.02 (2.51)	10.522	<.001	0.774
Hinting T2	377	14.31 (3.50)	287	16.56 (2.48)	9.696	<.001	0.726
Original scoring							
Hinting T1	395	15.63 (3.41)	299	17.92 (2.02)	11.039	<.001	0.791
Hinting T2	377	16.10 (3.39)	287	18.30 (1.88)	10.660	<.001	0.775

Early psychosis sample	*n*	*M* (*SD*)	*n*	*M* (*SD*)			
SCOPE scoring							
Hinting T1	38	15.82 (2.82)	39	17.72 (1.78)	3.533	.001	0.808
Hinting T2	36	17.08 (2.13)	36	18.00 (1.64)	2.047	.044	0.484
Original scoring							
Hinting T1	38	17.61 (2.40)	39	18.69 (1.06)	2.561	.013	0.585
Hinting T2	36	18.44 (1.75)	36	18.83 (1.56)	.997	.322	0.235

Abbreviation: SCOPE, Social Cognition Psychometric Evaluation; SSPA, Social Skills Performance Assessment.

#### 3.5.2 Early psychosis sample

Similar to the chronic sample, patients and healthy controls significantly differed on task performance when utilizing the original scoring criteria at visit 1 yet failed to meet significance at visit 2. Utilizing the updated SCOPE scoring system resulted in larger effect sizes at the initial visit and significantly differentiated between patient and control samples at retest.

## 4 DISCUSSION

The current study aimed to examine the psychometric properties of the Hinting task when using stricter scoring criteria developed as part of the SCOPE study, compared to the same data scored with the original scoring system. Chronic patients and early psychosis patients were analyzed separately to determine if the psychometric properties differed according to stage of illness. Although our overall results are mixed, the more stringent SCOPE criteria addressed key concerns regarding the Hinting task, namely reducing ceiling effects and better differentiating between patients and controls in an early psychosis sample; however, the updated criteria failed to significantly improve other psychometric properties. Further, we observed unique improvements in the relationship between performance and outcome measures when implementing a stricter scoring system, which may have added clinical utility when using this task.

Overall, SCOPE scoring criteria significantly lowered the mean scores for all examined groups, and the number of participants scoring at ceiling for the task was greatly reduced when implementing the SCOPE scoring across all samples. Importantly, the SCOPE criteria still differentiated patient and control groups for the chronic sample in a manner that was highly similar to the original scoring system, and significantly differentiated between early psychosis patients and matched controls with larger effect sizes. As the presence of ceiling effects has been raised as one of the primary criticisms against the Hinting task, particularly in relation to its suitability for use in clinical trials, these findings suggest that the revised SCOPE criteria may partially remedy this problem. Relatedly, applying the more stringent scoring system did not appear to disproportionately impact patient scores relative to those of healthy controls.

Results were not uniformly positive however, and within the chronic sample, SCOPE scoring resulted in mixed impacts on psychometric properties of the task. Specifically, SCOPE scoring resulted in test‐retest reliability within the acceptable range despite a statistically significant decrease relative to the original scoring for the chronic patient sample. For healthy controls, scoring criteria did not impact test‐retest reliability, and both scoring systems resulted in estimates below the desired range. Estimates of internal consistency did not meet recommended levels for either scoring criteria, and no significant improvement was seen using one scoring system over the other. Additionally, we only observed small practice effects on performance from initial to follow‐up visits, using either scoring criteria.

Importantly, correlations between Hinting total scores at the initial visit and functional capacity were significantly improved using SCOPE scoring, and when controlling for neurocognitive ability, we observed a similar increase in the relationship between informant rated daily functioning and hinting performance. It is unsurprising that neurocognitive ability may partially account for the significant increase in the relationship between SCOPE scoring and functional capacity, as SCOPE scoring may tap into problem solving aspects of financial and communication skills that are assessed in the UPSA. However, the uniquely significant relationship between hinting performance and informant reports of daily functioning may indicate that SCOPE scoring is measuring unique aspects of social ability/understanding that is related to interpersonal interactions above and beyond just neurocognitive ability.

The results for our early psychosis sample similarly indicate that the SCOPE criteria provided some unique benefits with minimal costs to the psychometric properties of the task. The most notable observation within this sample was that SCOPE criteria better differentiated between patients and controls, with rather large effects at the initial visit and smaller, albeit significant effects, at follow‐up. As with the chronic sample, both scoring methods resulted in test‐retest reliability within the acceptable range for the patient sample, whereas estimates for healthy controls were below cut‐offs. We did not observe any significant impact of scoring system on test‐retest reliability across groups. Although Cronbach's alpha was below desired levels for all groups and time points, we did observe a significant improvement in internal consistency using SCOPE scoring criteria for healthy controls at the initial visit. As noted above, this may be due to the limited variability in item responses within this sample, and ways to improve this aspect of the task are discussed below. Additionally, we observed an increase in practice effects for the early psychosis patient sample, with moderate effects when using the SCOPE criteria compared to small effects observed in the original criteria. Notably, we did not see any significant increased relationship between hinting performance and outcome measures when using either scoring criteria.

The different pattern of results in our early psychosis sample indicates that age or stage of illness may impact some of the psychometric properties of the SCOPE scoring. As noted in Ludwig et al., (2017), it is plausible that patients early in the course of illness may either retain levels of premorbid functioning or exhibit reduced deficits in ToM, impacting some of our results (i.e., increased practice effects, or nonsignificant associations between occupational skills and performance on the Hinting task). The current results may also be confounded by the fact that our early psychosis sample was significantly smaller than the chronic sample, as well as younger, more educated, and scoring higher on a measure of premorbid IQ. Taken together, these findings suggest that that task's ability to detect improved performance may be restricted for some individuals, and that researchers may need to assess potential costs and benefits to utilizing the Hinting Task along with the SCOPE scoring system in patient samples with attenuated social cognitive deficits and healthy samples.

It is also important to note that even though SCOPE scoring reduced the total number of those with near‐perfect scores, these results highlight a potential inherent limitation of the task. In the chronic sample, 24% of patients and 47% of controls scored in the near‐perfect range, and over 50% of the early psychosis patients and controls scored in the near‐perfect range even with the more restrictive SCOPE scoring criteria. Although we believe that the SCOPE scoring system addresses key concerns with using the Hinting Task in its current form, there are several avenues for researchers to further improve the psychometric properties of the Hinting Task, especially for use in healthy and more normative patient samples. Specifically, researchers could create and test alternate vignettes to either add to the current task and increase total number of items, or to replace items that perform poorly to increase internal consistency and construct validity. This work would also benefit from the creation of an alternate form of the current task for use in clinical trials. Researchers could also expand the rating scale beyond the 0–2 rating per item to provide more nuances in subject response; however, this would greatly impact the ease with which the task can currently be administered. Finally, other suggested improvements would be to standardize the task through electronic/digital methods to reduce the interrater variability; however, this could be challenging given the task's current open‐ended prompt structure. Although SCOPE scoring does not address all the challenges associated with the Hinting task, we believe it provides incremental benefits that warrant adoption until more substantial task improvements can be validated and peer reviewed.

In summary, the present study demonstrated that the SCOPE scoring criteria improves key psychometric properties of the Hinting task and increased relationships with outcome measures when administered to persons diagnosed with a schizophrenia spectrum disorder. Stricter scoring, as demonstrated by lowered group means and reductions in near‐perfect scores on the task, allows for more variability not only within patient samples but also within healthy samples. As such, employing the more stringent scoring criteria from the SCOPE study may lead to more accurate assessment of ToM deficits and reduce potential statistical violations when directly comparing performance between clinical and non‐clinical samples. Limiting ceiling effects increases the utility of the Hinting task in clinical and research settings. Further, stronger relationships with outcome measures indicate a more precise measurement of functionally important aspects of ToM, thus arguably resulting in a stronger tool for clinical research. It is important to note that test‐retest reliability and internal consistency decreased in some samples with use of the SCOPE scoring system, and as such, should be taken into consideration when using the stricter scoring system. Despite these limitations, this study clarifies and emphasizes that the SCOPE study endorsement of the Hinting task as acceptable for use in clinical trials carries the caveat that the more stringent scoring should be used. As such, we strongly recommend a wider adoption of the revised scoring system when using the Hinting task.

## CONFLICT OF INTEREST

The authors declare no conflicts of interest.

## Supporting information


**Appendix** **S1.** Supporting information.
