# Supplementary material for: Measuring mentalizing: A comparison of scoring methods for the hinting task
Source: Int J Methods Psychiatr Res. 2020 May 9;29(2):e1827. doi: 10.1002/mpr.1827 (PMC7301277; doi:10.1002/mpr.1827)
Supplement: Supplementary file 1 — Appendix S1. Supporting information. [file MPR-29-e1827-s001.docx]

Supplemental material for

Measuring mentalizing: A comparison of scoring methods for the Hinting Task

Hans Klein, M.S., Cassi Springfield, M.S., Emily Bass, B.A., Kelsey Ludwig, M.A, David Penn, Ph.D., Philip Harvey, Ph.D., & Amy Pinkham, Ph.D.

1. SCOPE Hinting Task scoring guidelines

The scoring guidelines utilized in the SCOPE study are provided on pages 3 through 5.

1. Correlations between Hinting scorings and neurocognition.

Table 1 presents the correlations between Hinting scores and MCCB domain scores.

1. Breakdown of analyses by phases of SCOPE

In order to improve interpretability of results as they relate to previous publications of the SCOPE study, analyses were repeated using the sample breakdown from each phase of SCOPE. Please note that SCOPE results listed may differ slightly from published results as six participants’ data that were included in the published analyses were not available to be recoded and therefore, those individuals are not included in these supplemental analyses. Table 2 presents the demographic and clinical characteristics for each phase of SCOPE, while Tables 3 - 6 present the psychometric properties of each sample using both the SCOPE scoring and the original scoring criteria. Comparisons of performance between patients and healthy controls are listed in Table 7. For ease of reference, SCOPE phase 3 (SCOPE 03) utilizes SCOPE scoring data published in Pinkham et al. (2016), and SCOPE phase 5 (SCOPE 05) utilizes SCOPE scoring data published in Pinkham et al. (2018). SCOPE phase 4 (SCOPE 04) utilizes a previously unpublished dataset that was generated from the modifications phase of the larger project.

1. Supplemental analyses of early psychosis sample

Tables 8 – 12 present the demographics and psychometric analyses for the early psychosis sample when excluding the three outliers noted in the main article. In the early psychosis sample when outliers are excluded, patients and healthy controls were relatively similar on age and premorbid IQ, however, the two groups differed significantly on years of education, *t*(72) = 3.524, *p* = .001, *d* = .823. Analyses of group differences are listed in Table 13. Results mirror that of the primary analyses, with SCOPE scoring criteria better discriminating between patients and healthy controls on performance of the task at both timepoints.

**Hinting Task – SCOPE Scoring Criteria and Guidelines**

**Hinting Item Scoring: 2 = Correct answer with first hint**

**1 = Correct answer with second hint**

**0 = Paraphrase of what character said or No response**

**20 = Total possible points**

*Notes on scoring:*

- *If the respondent gives a* ***1 point*** *response on the first hint, you may score a* ***1 and move on to the next question.*** *If respondent gives a* ***0 point*** *response on first hint,* ***present the second hint****.*
- *You may re-read a story once if the respondent requests it.*
- Think of scoring more in terms of ***key elements or words*** that must be present rather than precisely matching the phrases listed as the correct answer on the score sheet.
  - For example, on 10 the key element is that Patsy wants John’s help with the suitcases. You can give the following response a score of 2: *“She’s struggling with them and it would be John’s call of duty to help her.”*

** A list of **common responses** has been added in **bold** to help standardize scoring. Please read this guide every time you score to insure reliability. Please note that Item 6 has extensive scoring guidelines, so additional response examples have been added to this section.

1. **Required components for a 2-point answer:**

- Respondent must mention *wanting something to drink* **or** that George is *tired* ***and*** *wants to rest* *before talking* for 2 points.

Common 2 point responses:

**He wants something to drink.**

**He’s tired and doesn’t want to talk business immediately.**

Common response with skip logic:

If there’s no mention of wanting a drink or that he’s tired and needs a break first,

present the second hint. If respondent states that **George is tired; George needs a**

**break;** **He’s not ready to talk** after the first hint, score a 1 and move on (do not

award any points for this response after the second hint).

Other Common responses:

**He wants to relax and cool down** = give second hint or if this response is given on second

hint, score 0 points

*2*. **Required components for a 2-point answer:**

- *Subject/verb* (She (Melissa) wants…; She (Anne) should…; Please (you) clean…)
- The idea that Anne *wants Melissa/her specifically to do/have done it*
- Using the word “*clean*” (or a synonym)

Common 2 point responses:

**She should have cleaned the tub out.**

**“Please clean the bathtub!”**

**She wants her to clean it**

Other Common responses:

**Clean it** = give second hint or if this response is given on second hint, score 1 point

**Pick up after herself** = give second hint or if this response is given on second hint, score 0

points

*3*. Common 2 point responses:

**He wants the Twinkies.**

**“Can you buy me some, mom?”**

Other Common responses:

**He wants some; He wants to eat one; He is hungry; Feed him** = give second hint or if any

of these responses are given on second hint, score 0 points

4. **Required components for a 2-point answer:**

- *Subject/verb* (He wants…; Would you…?)
- The idea that he *wants* *Jane/her specifically* to do it
- Using the word “*iron*” or “*press*”

Common 2 point responses:

**He wants her to iron the shirt for him.**

**“Would you iron it for me, please?”**

Other Common responses:

**Iron it; Press it** = give second hint or if this response is given on second hint, score 1 point

5. **Required components for a 2-point answer:**

- *Subject/verb* (She wants…)
- The idea that she *wants David/him specifically* to do it
- *Money component* (paying for her, treating her, giving/lending money, etc.)
- *Ideal but not required for 2 points*: the concept of *taking her out/going out*

Common 2 point responses:

**She wants him to lend her some money**

**She wants him to take her out and pay for it.**

**“Could you pay for me to go out tonight?”**

Other Common responses:

**Take her out; Take her to dinner; Pay for her** = give second hint or if any of these

responses are given on second hint, score 1 point

**Give her the money** = give second hint or if this response is given on second hint, score 0

points

6. Common 2 point responses:

**He wants to do the project.**

**“Could you give me the project instead?”**

**He wants Richard to consider giving it/the project to him.**

**He wants the job/project.**

**“Give me the project/job.”**

Other Common responses:

**Give it to him; Let him do it** = give second hint or if either response is given on second hint,

score 1 point

If respondent says something vague like **He wants it;** **He wants to do it; He’s available** = give second hint or if this response is given on second hint, score 0 points

7. **Required components for a 2-point answer:**

- Using the word “*puppy*” or “*dog*”
- The idea of it being *for her birthday* ***and/or*** *her dad getting it for her*. (e.g. “She wants a puppy/dog for her birthday/from her dad.”)

Common 2 point responses:

**She wants a dog for her birthday.**

**“Dad will you get me a puppy/dog?”**

Common response with skip logic:

**She wants a dog/pet/animal** after the first hint score a 1 and move on.

8. **Required components for a 2-point answer:**

- *Subject/verb* (She wants…; Can you…?)
- The idea that Betsy *wants Michael/him specifically* to do it
- Use of the word “*shelves*”
- Must include the concept of *putting up the shelves/ assembling shelves*, not just unpacking them.
- ***The only exception to this is verbatim: “She wants to put the glassware on the shelves.***”

Common 2 point responses:

**She wants to put the glassware on the shelves.**

**“Can you put the shelves up?”**

Common response with skip logic:

If respondent says “**She wants him to unpack the shelves**” score a 1 and move on.

Other Common responses:

**“Unpack them”** = give second hint or if this response is given on second hint, score 1 point

**Help her unpack**; **Do it himself** = give second hint or if either response is given on second

hint, score 0 points

9. Common 2 point responses:

**She wants to trade trains with him.**

**“Can I have the red train?”**

**She wants the red one.**

Other Common responses:

**Give it to her**; **Give her the blue train** = give second hint or if either response is given on

second hint, score 0 points

10. **Required components for a 2-point answer:**

- *Subject/verb* (She wants…; Can you…?)
- The idea that she wants *John/him specifically* to do it
- The concept of *helping* ***and/or*** *carrying the suitcases/them*

Common 2 point responses:

**She wants him to help her with the suitcases**

**“Could you help me out here, please?”**

Other Common responses:

**Help her; Help carry them** = give second hint or if this response is given on second hint,

score 1 point

**Take them; Take one of them** = give second hint or if any of these responses are given on

second hint, score 0 points

|  | SCOPE Scoring |  | Original Scoring |  |
| --- | --- | --- | --- | --- |
|  | Hinting T1 | Hinting T2 | Hinting T1 | Hinting T2 |
| Chronic Subset | *n* =384 | *n* =376 | *n* =384 | *n* =376 |
| Neurocognitive |  |  |  |  |
| Trails A | -.108^*^ | -.199^*^ | -.049 | -.095 |
| Symbol Coding | .202^***^ | .271^***^ | .184^***^ | .233^***^ |
| HVLT -R | .249^***^ | .276^***^ | .174^***^ | .207^***^ |
| Letter-Number Span | .353^***^ | .402^***^ | .191^***^ | .235^***^ |
| Animal Naming | .159^***^ | .172^***^ | .115^*^ | .116^*^ |
| Early Psychosis Subset | *n* =38 | *n* =36 | *n* =38 | *n* =36 |
| Neurocognitive |  |  |  |  |
| Trails A | -.115 | -.039 | -.052 | -.113 |
| Symbol Coding | -.120 | -.181 | -.207 | -.099 |
| HVLT -R | .157 | .249 | .179 | .179 |
| Letter-Number Span | .170 | .280 | .151 | .293 |
| Animal Naming | .261 | .173 | .209 | .181 |
| ^*^*p* ≤ .05; ^**^ *p* ≤ .01; ^***^ *p* ≤ .001 | | | | |

Table 1. Correlations between initial visit Hinting scores and neurocognitive domains in patients.

Table 2.

Participant demographic and clinical characteristics for individual samples collected during each phase of SCOPE.

|  | SCOPE 03 | | | SCOPE 04 | | | SCOPE 05 | | |
| --- | --- | --- | --- | --- | --- | --- | --- | --- | --- |
|  | Patients (*n*=178) | Controls (*n*=104) | Patients (*n*=56) | | Controls (*n*=47) | Patients (*n*=217) | | Controls (*n*=153) |  |
|  | *n*(%) | *n*(%) | *n*(%) | | *n*(%) | *n*(%) | | *n*(%) |  |
| Male | 117 (65.7) | 49 (47.1) | 30 (53.6) | | 28 (59.6) | 141 (65.0) | | 96 (62.7) |  |
| Race |  |  |  | |  |  | |  |  |
| Caucasian | 76 (42.7) | 43 (41.3) | 20 (35.7) | | 17 (36.2) | 114 (52.5) | | 80 (52.3) |  |
| African American | 93 (52.2) | 55 (52.9) | 31 (55.4) | | 28 (59.6) | 87 (40.1) | | 61 (39.9) |  |
| Asian | 4 (2.2) | 4 (3.8) | 2 (3.6) | | 2 (4.3) | 6 (2.8) | | 4 (2.6) |  |
| Other | 5 (2.8) | 2 (1.9) | 3 (5.4) | | 0 (0.0) | 10 (4.6) | | 8 (5.2) |  |
| Ethnicity |  |  |  | |  |  | |  |  |
| Hispanic | 38 (21.3) | 21 (20.2) | 11 (19.6) | | 13 (27.7) | 33 (15.2) | | 25 (16.3) |  |
| Non-Hispanic | 140 (78.7) | 83 (79.8) | 45 (80.4) | | 34 (72.3) | 184 (84.8) | | 128 (83.7) |  |
|  | M(SD) | M(SD) | M(SD) | | M(SD) | M(SD) | | M(SD) |  |
| Age | 41.86 (12.26) | 39.20 (13.70) | 43.29 (10.16) | | 42.62 (9.61) | 41.72 (11.67) | | 41.84 (12.39) |  |
| Education (years)^*^ | 12.70 (2.14) | 13.36 (1.66) | 12.94 (2.45) | | 14.44 (1.85) | 13.03 (2.48) | | 14.17 (1.90) |  |
| WRAT-3^*^ | 93.65 (15.92) | 95.35 (13.19) | 92.96 (14.52) | | 99.28 (11.25) | 94.73 (14.65) | | 101.14 (11.51) |  |
| UPSA-B | 69.76 (14.28) | - | 72.26 (14.57) | | - | 70.68 (14.12) | | - |  |
| SSPA-Avg | 4.12 (0.55) | - | 4.26 (0.45) | | - | 4.13 (0.55) | | - |  |
| SLOFinf-Avg | 3.96 (0.56) | - | 4.16 (0.57) | | - | 4.12 (0.64) | | - |  |
| SLOFsr-Avg | - | - | 4.44 (0.37) | | - | 4.12 (0.58) | | - |  |
| PANSS (Initial) |  |  |  | |  |  | |  |  |
| Positive total | 16.16 (5.78) | - | 16.13 (4.98) | | - | 15.96 (5.32) | | - |  |
| Negative total | 13.72 (5.29) | - | 12.68 (3.31) | | - | 14.04 (5.64) | | - |  |
| General total | 30.82 (7.90) | - | 31.25 (7.90) | | - | 31.60 (8.10) | | - |  |
| PANSS (Follow up) |  |  |  | |  |  | |  |  |
| Positive total | 15.38 (5.05) | - | 16.26 (5.22) | | - | 15.57 (5.37) | | - |  |
| Negative total | 13.54 (5.14) | - | 13.85 (5.37) | | - | 13.89 (5.12) | | - |  |
| General total | 29.48 (7.52) | - | 30.81 (7.25) | | - | 30.43 (7.65) | | - |  |

^*^ Group differences for each phase of SCOPE. For SCOPE03, groups differed only on years of education, *t*(280) = 2.677, *p* = .001, *d* = 0.334; For SCOPE 04, groups differed on years of education, t(101) = 3.447, *p* = .001, *d* =0.683, and WRAT-3, *t*(100.397) = 2.485, *p* = .015, *d* = 0.481. Similarly, for SCOPE 05, groups differed on years of education, *t*(368) = 4.817, *p* < .001, *d* = 0.505 and WRAT-3, *t*(363.624) = 4.710, *p* < .001, *d* = 0.477

Table 3.

Test-retest reliability and internal consistency for individual samples collected during each phase of SCOPE.

|  | Test-Retest Reliability  (Pearson *r*) | | Internal Consistency  (Cronbach’s Alpha) | | | |
| --- | --- | --- | --- | --- | --- | --- |
| SCOPE 03 |  |  |  |  |  |  |
| Task | Patients  *n* = 169 | Controls  *n* = 97 | Patients T1  *n* = 177 | Patients T2  *n* = 170 | Controls T1  *n* = 103 | Controls T2  *n* = 98 |
| Hinting  *(SCOPE Scoring)* | .643 | .573 | .731 | .720 | .563 | .427 |
| Hinting  (*Original Scoring)* | .730 | .572 | .742 | .722 | .532 | .509 |
| Significance test^†^ | -2.153^*^ | 0.012 | 0.101 | 0.004 | 0.124 | 0.634 |
| SCOPE 04 |  |  |  |  |  |  |
| Task | Patients  *n* = 53 | Controls  *n* = 46 | Patients T1  *n* = 56 | Patients T2  *n* = 53 | Controls T1  *n* = 47 | Controls T2  *n* = 46 |
| Hinting  *(SCOPE Scoring)* | .583 | .386 | .553 | .660 | .461 | .419 |
| Hinting  (*Original Scoring)* | .701 | .155 | .611 | .727 | .303 | .279 |
| Significance test^†^ | -1.173 | 1.862 | 0.272 | 0.841 | 0.970 | 0.547 |
| SCOPE 05 |  |  |  |  |  |  |
| Task | Patients  *n* = 206 | Controls  *n* = 147 | Patients T1  *n* = 216 | Patients T2  *n* = 207 | Controls T1  *n* = 153 | Controls T2  *n* = 147 |
| Hinting  *(SCOPE Scoring)* | .696 | .617 | .682 | .771 | .629 | .714 |
| Hinting  (*Original Scoring)* | .787 | .734 | .730 | .733 | .681 | .737 |
| Significance test^†^ | -2.401^*^ | -2.468^*^ | 1.646 | 1.570 | 1.162 | 0.426 |
| *^*^p* < .05; ^**^ *p* < .01;  ^†^ Fisher’s z was calculated to compare test-retest reliability estimates. Feldt tests were performed to compare estimates of internal consistency. | | | | | | |

Table 4.

Utility as a repeated measure for individual samples collected during each phase of SCOPE.

|  | T_1_ | | | T_2_ | | | T_2_ – T_1_ Difference | | Number at Floor/Ceiling | |  |  |  |
| --- | --- | --- | --- | --- | --- | --- | --- | --- | --- | --- | --- | --- | --- |
| SCOPE 03 |  |  |  |  |  |  |  |  |  |  |  |  |  |
| Task | Mean | *SD* | Skew/Kurtosis | Mean | *SD* | Skew/Kurtosis | Mean | *SD* | T_1_ | T_2_ | *t* | *p* | Cohen’s *d*_z_ |
| Patients |  |  |  |  |  |  |  |  |  |  |  |  |  |
| Hinting (*n* = 170)  (SCOPE Scoring) | 13.65 | 3.81 | -.688/-.210 | 14.25 | 3.63 | -.924/.715 | 0.60 | 3.15 | 0/2 | 0/2 | 2.494 | .014 | 0.191 |
| Hinting (*n* = 169)  (Original Scoring) | 15.37 | 3.75 | -.804/-.239 | 15.97 | 3.60 | -1.090/.990 | 0.60 | 2.71 | 0/17 | 0/26 | 2.871 | .005 | 0.221 |
| Controls |  |  |  |  |  |  |  |  |  |  |  |  |  |
| Hinting (*n* = 97)  (SCOPE Scoring) | 16.74 | 2.28 | -1.373/3.965 | 17.35 | 1.83 | -2.092/9.566 | 0.61 | 1.94 | 0/6 | 0/7 | 3.089 | .003 | 0.314 |
| Hinting (*n* = 97)  (Original Scoring) | 17.86 | 2.09 | -1.981/5.280 | 18.33 | 1.89 | -2.146/6.008 | 0.47 | 1.85 | 0/19 | 0/27 | 2.526 | .013 | 0.256 |
| SCOPE 04 |  |  |  |  |  |  |  |  |  |  |  |  |  |
| Task | Mean | *SD* | Skew/Kurtosis | Mean | *SD* | Skew/Kurtosis | Mean | *SD* | T_1_ | T_2_ | *t* | *p* | Cohen’s *d*_z_ |
| Patients |  |  |  |  |  |  |  |  |  |  |  |  |  |
| Hinting (*n* = 53)  (SCOPE Scoring) | 13.55 | 3.31 | -.695/.121 | 14.83 | 3.40 | -.976/.867 | 1.28 | 3.07 | 0/0 | 0/1 | 3.047 | .004 | 0.419 |
| Hinting (*n* = 53)  (Original Scoring) | 15.58 | 3.18 | -.943/.110 | 16.17 | 3.58 | -.741/-.673 | 0.58 | 2.64 | 0/2 | 0/10 | 1.612 | .113 | 0.221 |
| Controls |  |  |  |  |  |  |  |  |  |  |  |  |  |
| Hinting (*n* = 46)  (SCOPE Scoring) | 16.41 | 2.11 | -.766/1.883 | 16.76 | 2.11 | -.635/.043 | 0.35 | 2.34 | 0/2 | 0/3 | 1.008 | .319 | 0.149 |
| Hinting (*n* = 46)  (Original Scoring) | 18.54 | 1.39 | -1.123/1.625 | 18.67 | 1.35 | -1.236/1.877 | 0.13 | 1.78 | 0/14 | 0/15 | 0.496 | .622 | 0.073 |
| SCOPE 05 |  |  |  |  |  |  |  |  |  |  |  |  |  |
| Task | Mean | *SD* | Skew/Kurtosis | Mean | *SD* | Skew/Kurtosis | Mean | *SD* | T_1_ | T_2_ | *t* | *p* | Cohen’s *d*_z_ |
| Patients |  |  |  |  |  |  |  |  |  |  |  |  |  |
| Hinting (*n* = 206)  (SCOPE Scoring) | 13.42 | 3.71 | -.781/.626 | 13.89 | 4.11 | -1.000/.736 | 0.48 | 3.07 | 1/2 | 1/4 | 2.222 | .027 | 0.155 |
| Hinting (*n* = 206)  (Original Scoring) | 14.64 | 3.89 | -.684/-.243 | 15.03 | 3.86 | -.794/.299 | 0.39 | 2.53 | 0/11 | 0/19 | 2.206 | .029 | 0.154 |
| Controls |  |  |  |  |  |  |  |  |  |  |  |  |  |
| Hinting (*n* = 147)  (SCOPE Scoring) | 15.18 | 3.01 | -1.256/2.874 | 15.65 | 3.31 | -1.308/2.258 | 0.47 | 2.78 | 0/7 | 0/12 | 2.049 | .042 | 0.169 |
| Hinting (*n* = 147)  (Original Scoring) | 17.50 | 2.61 | -2.077/5.722 | 17.86 | 2.67 | -2.381/7.156 | 0.37 | 1.93 | 0/25 | 0/38 | 2.311 | .022 | 0.191 |

Table 5.

Paired samples statistical test comparing Hinting task scoring methods for individual samples collected during each phase of SCOPE.

| SCOPE 03 |  |  |  |  | |  |
| --- | --- | --- | --- | --- | --- | --- |
| Task | Original *M(SD)* | SCOPE *M(SD)* | *t (paired samples)* | *p* | Cohen’s *d_z_* | |
| Patients |  |  |  |  |  | |
| Hinting T1 (*n* = 177) | 15.24 (3.87) | 13.59 (3.88) | 7.247 | < .001 | 0.545 | |
| Hinting T2 (*n* = 170) | 15.92 (3.66) | 14.22 (3.64) | 7.610 | < .001 | 0.584 | |
| Controls |  |  |  |  |  | |
| Hinting T1 (*n* = 103) | 17.88 (2.06) | 16.72 (2.30) | 5.167 | < .001 | 0.509 | |
| Hinting T2 (*n* = 98) | 18.35 (1.88) | 17.35 (1.82) | 5.110 | < .001 | 0.516 | |
| SCOPE 04 |  |  |  |  |  | |
| Task | Original *M(SD)* | SCOPE *M(SD)* | *t (paired samples)* | *p* | Cohen’s *d_z_* | |
| Patients |  |  |  |  |  | |
| Hinting T1 (*n* = 56) | 15.71 (3.15) | 13.57 (3.23) | 4.796 | < .001 | 0.641 | |
| Hinting T2 (*n* = 53) | 16.17 (3.58) | 14.83 (3.40) | 3.078 | .003 | 0.423 | |
| Controls |  |  |  |  |  | |
| Hinting T1 (*n* = 47) | 18.57 (1.39) | 16.43 (2.09) | 8.833 | < .001 | 1.288 | |
| Hinting T2 (*n* = 46) | 18.67 (1.35) | 16.76 (2.11) | 6.804 | < .001 | 1.003 | |
| SCOPE 05 |  |  |  |  |  | |
| Task | Original *M(SD)* | SCOPE *M(SD)* | *t (paired samples)* | *p* | Cohen’s *d_z_* | |
| Patients |  |  |  |  |  | |
| Hinting T1 (*n* = 216) | 14.66 (3.86) | 13.35 (3.72) | 5.172 | < .001 | 0.352 | |
| Hinting T2 (*n* = 207) | 15.02 (3.85) | 13.87 (4.12) | 4.618 | < .001 | 0.321 | |
| Controls |  |  |  |  |  | |
| Hinting T1 (*n* = 153) | 17.52 (2.58) | 15.18 (3.00) | 11.927 | < .001 | 0.964 | |
| Hinting T2 (*n* = 147) | 17.86 (2.67) | 15.65 (3.31) | 11.199 | < .001 | 0.924 | |

Table 6.

Correlations between initial visit Hinting scores and functional outcome measures for individual samples collected during each phase of SCOPE.

|  | UPSA Total | SSPA Average | SLOF Informant | SLOF Self-Report |
| --- | --- | --- | --- | --- |
| SCOPE 03 | *n* =176 | *n* =175 | *n* =175 | *n* = NA |
| Hinting  *(SCOPE Scoring)* | .454^***^ (.284^***^) | .387^***^ (.262^***^) | .175^*^ (.086) | - |
| Hinting  *(Original Scoring)* | .308^***^ (.216^**^) | .382^***^ (.279^***^) | .218^**^ (.175^*^) | - |
| Fisher’s z | 2.748^**^ (1.192) | .092 (-.298) | -.739 (-1.517) | - |
| Neurocognitive |  |  |  | - |
| Trails A | -.272^***^ | -.100 | -.235^***^ | - |
| Symbol Coding | .269 | .306^***^ | .270^***^ | - |
| HVLT -R | .446^***^ | .370*** | .132 | - |
| Letter-Number Span | .551^***^ | .318^***^ | .237^**^ | - |
| Animal Naming | .188^*^ | .170^*^ | .110 | - |
| SCOPE 04 | *n* =56 | *n* =56 | *n* =55 | *n* =10 |
| Hinting  *(SCOPE Scoring)* | .366^**^ (.319^*^) | .300^*^ (.230) | .161 (.164) | .029 (-.059) |
| Hinting  *(Original Scoring)* | .099 (.031) | .093 (.055) | .119 (.187) | -.158 (-.346 ) |
| Fisher’s z | 1.993 (2.126*) | 1.506 (1.250) | .293 (-.162) | .481 (.776) |
| Neurocognitive |  |  |  |  |
| Trails A | -.270^*^ | -.197 | -.222 | -.496 |
| Symbol Coding | .465^***^ | .227 | .191 | -.266 |
| HVLT -R | .442^***^ | .393^**^ | .281^*^ | .192 |
| Letter-Number Span | .482^***^ | .035 | .079 | .065 |
| Animal Naming | .174 | .193 | -.107 | .071 |
| SCOPE 05 | *n* =206 | *n* =208 | *n* =133 | *n* =176 |
| Hinting  *(SCOPE Scoring)* | .404^***^ (.272^***^) | .437^***^ (.341^***^) | .193^*^ (.124) | -.071 (-.107) |
| Hinting  *(Original Scoring)* | .249^***^ (.160^*^) | .308^***^ (.246^***^) | .092 (.050) | -.043 (-.056) |
| Fisher’s z | 2.450^*^ (1.686) | 2.090^*^ (1.474) | 1.194 (.866) | -.376 (-.687) |
| Neurocognitive |  |  |  |  |
| Trails A | -.291^***^ | -.213^**^ | .022 | .022 |
| Symbol Coding | .388^***^ | .286^***^ | .097 | -.018 |
| HVLT -R | .393^***^ | .337^***^ | .198^*^ | -.010 |
| Letter-Number Span | .424^***^ | .318^***^ | .219^*^ | .143 |
| Animal Naming | .235^***^ | .194^**^ | .043 | -.067 |
| Note: Correlations listed in parentheses are partial correlations between initial visit Hinting scores and functional outcome measures in patients after controlling for neurocognitive ability as measured by MATRICS consensus cognitive battery (MCCB) subscales. Fisher’s z calculated to compare the effect of scoring criterion on correlations with functional outcome measures.  ^*^*p* ≤ .05; ^**^ *p* ≤ .01; ^***^ *p* ≤ .001 | | | | |

Table 7.

Group differences on Hinting Task for individual samples collected during each phase of SCOPE.

|  |  |  |  |  |  |  | |  |
| --- | --- | --- | --- | --- | --- | --- | --- | --- |
| Task | Patients | | Controls | | *t* | *p* | Cohen’s *d* | |
| SCOPE 03 | *n* | *M (SD)* | *n* | *M (SD)* |  |  |  | |
| SCOPE Scoring |  |  |  |  |  |  |  | |
| Hinting T1 | 177 | 13.59 (3.88) | 103 | 16.72 (2.30) | 8.478 | < .001 | 0.924 | |
| Hinting T2 | 170 | 14.22 (3.64) | 98 | 17.35 (1.82) | 9.349 | < .001 | 1.009 | |
| Original Scoring |  |  |  |  |  |  |  | |
| Hinting T1 | 177 | 15.24 (3.87) | 103 | 17.88 (2.06) | 7.459 | < .001 | 0.795 | |
| Hinting T2 | 170 | 15.92 (3.66) | 98 | 18.35 (1.88) | 7.167 | < .001 | 0.776 | |
| SCOPE 04 | *n* | *M (SD)* | *n* | *M (SD)* |  |  |  | |
| SCOPE Scoring |  |  |  |  |  |  |  | |
| Hinting T1 | 56 | 13.57 (3.23) | 47 | 16.43 (2.09) | 5.339 | < .001 | 1.033 | |
| Hinting T2 | 53 | 14.83 (3.40) | 46 | 16.76 (2.11) | 3.444 | .001 | 0.671 | |
| Original Scoring |  |  |  |  |  |  |  | |
| Hinting T1 | 56 | 15.71 (3.15) | 47 | 18.57 (1.39) | 6.119 | < .001 | 1.141 | |
| Hinting T2 | 53 | 16.17 (3.58) | 46 | 18.67 (1.35) | 4.717 | < .001 | 0.900 | |
| SCOPE 05 | *n* | *M (SD)* | *n* | *M (SD)* |  |  |  | |
| SCOPE Scoring |  |  |  |  |  |  |  | |
| Hinting T1 | 216 | 13.35 (3.72) | 153 | 15.18 (3.00) | 5.226 | < .001 | 0.532 | |
| Hinting T2 | 207 | 13.87 (4.12) | 147 | 15.65 (3.31) | 4.510 | < .001 | 0.468 | |
| Original Scoring |  |  |  |  |  |  |  | |
| Hinting T1 | 216 | 14.66 (3.86) | 153 | 17.52 (2.58) | 8.511 | < .001 | 0.844 | |
| Hinting T2 | 207 | 15.02 (3.85) | 147 | 17.86 (2.67) | 8.194 | < .001 | 0.833 | |

Table 8.

Participant demographic and clinical characteristics for early psychosis sample when outliers are excluded.

|  | Early Psychosis sample |  |
| --- | --- | --- |
|  | Patients (*n*=37) | Controls (*n*=38) |
|  | *n*(%) | *n*(%) |
| Male | 32 (86.5) | 31 (81.6) |
| Race |  |  |
| Caucasian | 27 (73.0) | 25 (65.8) |
| African American | 4 (10.8) | 5 (13.2) |
| Asian | 2 (5.4) | 2 (5.3) |
| Other | 4 (10.8) | 6 (15.8) |
| Ethnicity |  |  |
| Hispanic | 2 (5.4) | 6 (15.8) |
| Non-Hispanic | 35 (94.6) | 32 (84.2) |
|  | M(SD) | M(SD) |
| Age | 23.59 (2.91) | 23.68 (3.39) |
| Education (years)^*^ | 14.07 (1.51) | 15.42 (1.82) |
| WRAT-3 | 105.76 (9.45) | 107.71 (9.00) |
| UPSA-B | 71.03 (11.42) | - |
| SSPA-Avg | 4.17 (0.39) | - |
| SLOFinf-Avg | 4.08 (0.63) | - |
| SLOFsr-Avg | 4.25 (0.47) | - |
| PANSS (Initial Visit) |  |  |
| Positive total | 17.59 (4.96) | - |
| Negative total | 16.54 (4.01) | - |
| General total | 36.14 (5.97) | - |
| PANSS (Follow up) |  |  |
| Positive total | 15.57 (4.05) | - |
| Negative total | 16.00 (4.40) | - |
| General total | 34.91 (6.71) | - |
| * Groups differed on only years of education, *t*(73) = 3.498, *p* = .001, *d* = .806. | | |

Table 9.

Test-retest reliability and internal consistency in early psychosis sample when outliers are excluded.

|  | Test-Retest Reliability  (Pearson *r*) | | Internal Consistency  (Cronbach’s Alpha) | | | |
| --- | --- | --- | --- | --- | --- | --- |
| Early Psychosis Subset |  |  |  |  |  |  |
| Task | Patients  (*n* = 35) | Controls  (*n* = 35) | Patients T1  (*n* = 37) | Patients T2  (*n* = 35) | Controls T1  (*n* = 38) | Controls T2  (*n* = 35) |
| Hinting  *(SCOPE Scoring)* | .644 | .191 | .601 | .395 | .535 | .181 |
| Hinting  (*Original Scoring)* | .318 | .258 | .480 | .320 | .149 | .015 |
| Significance test^†^ | 4.118^**^ | -0.529 | 1.382 | 0.180 | 4.906^*^ | 0.401 |
| ^*^ *p* < .05; ^**^ *p* < .01  ^†^ Fisher’s z was calculated to compare test-retest reliability estimates. Feldt tests were performed to compare estimates of internal consistency. | | | | | | |

Table 10.

Utility as a repeated measure in early psychosis sample when outliers are excluded.

|  | T_1_ | | | T_2_ | | | T_2_ – T_1_ Difference | | Number at Floor/Ceiling | |  |  |  | |
| --- | --- | --- | --- | --- | --- | --- | --- | --- | --- | --- | --- | --- | --- | --- |
| Early Psychosis subset | | | | | | | | | | | | | |  |
| Task | Mean | *SD* | Skew/Kurtosis | Mean | *SD* | Skew/Kurtosis | Mean | *SD* | T_1_ | T_2_ | *t* | *p* | Cohen’s *d_z_* |  |
| Patients | | | | | | | | | | | | | |  |
| Hinting (*n* = 35)  *(SCOPE Scoring)* | 16.09 | 2.48 | -.818/.426 | 17.26 | 1.88 | -1.179/2.194 | 1.17 | 1.92 | 0/1 | 0/2 | 3.615 | .001 | 0.611 |  |
| Hinting (*n* = 35)  *(Original Scoring)* | 17.97 | 1.79 | -1.292/1.216 | 18.63 | 1.37 | -.935/.103 | 0.66 | 1.88 | 0/5 | 0/11 | 2.070 | .046 | 0.350 |  |
| Controls | | | | | | | | | | | | | |  |
| Hinting (*n* = 35)  *(SCOPE Scoring)* | 17.94 | 1.55 | -1.101/1.666 | 18.20 | 1.13 | -1.449/4.749 | 0.26 | 1.74 | 0/4 | 0/3 | 0.875 | .388 | 0.148 |  |
| Hinting (*n* = 35)  *(Original Scoring)* | 18.77 | 1.06 | -.928/.936 | 19.06 | 0.80 | -.831/.842 | 0.29 | 1.15 | 0/9 | 0/10 | 1.467 | .152 | 0.248 |  |

Table 11.

Paired samples statistical test comparing Hinting task scoring methods in early psychosis sample when outliers are excluded.

| Early Psychosis sample |  |  |  |  |  |
| --- | --- | --- | --- | --- | --- |
| Task | Original M(SD) | SCOPE M(SD) | t (paired samples) | p | Cohen’s d_z_ |
| Patients |  |  |  |  |  |
| Hinting T1 (*n* = 37) | 17.86 (1.81) | 16.05 (2.44) | 7.404 | < .001 | 1.217 |
| Hinting T2 (*n* = 35) | 18.63 (1.37) | 17.26 (1.88) | 5.730 | < .001 | 0.969 |
| Controls |  |  |  |  |  |
| Hinting T1 (*n* = 38) | 18.74 (1.03) | 17.74 (1.80) | 4.616 | < .001 | 0.749 |
| Hinting T2 (*n* = 35) | 19.06 (0.80) | 18.20 (1.13) | 5.560 | < .001 | 0.940 |

|  | UPSA Total | SSPA Average | SLOF Informant | SLOF Self-Report |
| --- | --- | --- | --- | --- |
| Early Psychosis Subset | *n* = 37 | *n* = 37 | *n* = 30 | *n* = 37 |
| Hinting  *(SCOPE Scoring)* | .292 (.130) | .359^*^ (.391^*^) | -.234 (-.179) | .255^*^ (.292) |
| Hinting  *(Original Scoring)* | .328^*^ (.184) | .348^*^ (.309) | -.251 (-.213) | .169 (.198) |
| Fisher’s z | -.347 (-.499) | .107 (.809) | .142 (.282) | .809 (.894) |
| Neurocognitive |  |  |  |  |
| Trails A | -.266 | .107 | .100 | -.106 |
| Symbol Coding | .130 | .131 | .090 | .144 |
| HVLT-R | .574^***^ | .312 | -.115 | .160 |
| Letter-Number Span | .533^***^ | .482^**^ | .093 | .017 |
| Animal Naming | .310 | .398^*^ | -.017 | -.033 |
| Note: Correlations listed in parentheses are partial correlations between initial visit Hinting scores and functional outcome measures in patients after controlling for neurocognitive ability as measured by MATRICS consensus cognitive battery (MCCB) subscales. Fisher’s z calculated to compare the effect of scoring criterion on correlations with functional outcome measures.  ^*^*p* ≤ .05; ^**^ *p* ≤ .01; ^***^ *p* ≤ .001 | | | | |

Table 12.

Correlations between initial visit Hinting scores and functional outcome measures in early psychosis patients when outliers are excluded.

Table 13.

Group differences on Hinting Task in early psychosis patients when outliers are excluded.

| Task | Patients | | Controls | | *t* | *p* | Cohen’s *d* |
| --- | --- | --- | --- | --- | --- | --- | --- |
| Early Psychosis | *n* | *M (SD)* | *n* | *M (SD)* |  |  |  |
| SCOPE Scoring |  |  |  |  |  |  |  |
| Hinting T1 | 37 | 16.05 (2.44) | 38 | 17.74 (1.80) | 3.396 | .001 | 0.790 |
| Hinting T2 | 35 | 17.26 (1.88) | 35 | 18.20 (1.13) | 2.538 | .014 | 0.606 |
| Original Scoring |  |  |  |  |  |  |  |
| Hinting T1 | 37 | 17.86 (1.81) | 38 | 18.74 (1.03) | 2.551 | .013 | 0.600 |
| Hinting T2 | 35 | 18.63 (1.37) | 35 | 19.06 (0.80) | 1.594 | .117 | 0.383 |
